# Metabolic profiles alteration of Southern Thailand traditional sweet pickled mango during the production process

**DOI:** 10.3389/fnut.2022.934842

**Published:** 2022-09-08

**Authors:** Niken Indrati, Natthaporn Phonsatta, Patcha Poungsombat, Sakda Khoomrung, Punnanee Sumpavapol, Atikorn Panya

**Affiliations:** ^1^Food Microbiology and Safety Laboratory, Food Science and Technology Program, Faculty of Agro-Industry, Prince of Songkla University, Songkhla, Thailand; ^2^Food Biotechnology Research Team, Functional Ingredients and Food Innovation Research Group, National Center for Genetic Engineering and Biotechnology (BIOTEC), Thailand Science Park, Khlong Luang, Thailand; ^3^Metabolomics and Systems Biology, Department of Biochemistry, Faculty of Medicine Siriraj Hospital, Mahidol University, Bangkok, Thailand; ^4^Siriraj Metabolomics and Phenomics Center, Faculty of Medicine Siriraj Hospital, Mahidol University, Bangkok, Thailand; ^5^Department of Chemistry and Center of Excellence for Innovation in Chemistry (PERCH-CIC), Faculty of Science, Mahidol University, Bangkok, Thailand

**Keywords:** untargeted metabolomics profiling, sweet pickled mango, GC/Q-TOF, volatile metabolites, non-volatile metabolites, production processes

## Abstract

Sweet pickled mango named *Ma-Muang Bao Chae-Im* (MBC), a delicacy from the Southern part of Thailand, has a unique aroma and taste. The employed immersion processes (brining 1, brining 2, and immersion in a hypertonic sugar solution, sequentially) in the MBC production process bring changes to the unripe mango, which indicate the occurrence of metabolic profiles alteration during the production process. This occurrence was never been explored. Thus, this study investigated metabolic profile alteration during the MBC production process. The untargeted metabolomics profiling method was used to reveal the changes in volatile and non-volatile metabolites. Headspace solid-phase micro-extraction tandem with gas chromatography quadrupole time of flight (GC/QTOF) was employed for the volatile analysis, while metabolites derivatization for non-volatile analysis. In conclusion, a total of 82 volatile and 41 non-volatile metabolites were identified during the production process. Terpenes, terpenoids, several non-volatile organic acids, and sugars were the major mango metabolites that presented throughout the process. Gamma-aminobutyric acid (GABA) was only observed during the brining processes, which suggested the microorganism’s stress response mechanism to an acidic environment and high chloride ions in brine. Esters and alcohols were abundant during the last immersion process, which had an important role in MBC flavor characteristics. The knowledge of metabolites development during the MBC production process would be beneficial for product development and optimization.

## Introduction

Ma-Muang Bao Chae-Im (MBC) is a sweet pickled mango made from unripe endemic mango, found locally in the southern part of Thailand named *Ma-Muang Bao* (*Mangifera indica* L. Var.). Typically, *Ma-Muang Bao* has green color, sour taste, oblong shape, and relatively small size (3.5–4 cm × 4.5–5.5 cm), which is generally harvested around 60 days after pollination (1). According to a previous report by Maneerat and Meebun (2), it has low pH (2.47), indicating a high level of organic acid content. The osmotic dehydration technique is employed to produce MBC, which has a unique aroma and flavor. MBC is known to have a good-fermented aroma with a fruity note and sweet taste. These flavors were developed during the production process that requires several immersion processes.

In general, the production process of MBC requires several immersion processes using hypertonic solutions, i.e., brine and sugar solution. After the peeling, deseeding, and washing process, *Ma-Muang Bao* is then immersed in brine and sugar solution sequentially. Each immersion step takes 8 h to 2 days to complete the process. The brine is a high concentration of sodium chloride (∼ 6–10% w/v), whereas the sugar solution is about 30–50% (w/v) of sugar. In addition, the incorporation of calcium salt [CaCl_2_, CaCO_2_, Ca(OH)_2_] is employed to enhance the texture or firmness of an osmotically dehydrated product (3, 4).

When mango is submerged in a hypertonic solution, the multi-compound transfer takes place. The mass transfer from mango to the osmotic solution (leaching process) and from osmotic solution to mango (impregnation process) occurred throughout the immersion process. During this process, some organic acids, saccharides, fragrances, and water are leached into osmotic solutions, while salt and sugar from osmotic solutions infuse into the mango (5). The leaching out of solutes into osmotic solutions changed the volatile and non-volatile profiles of mango flesh. Compared to unripe mango, MBC had a less sour taste, and its sweetness was increased greatly due to lower organic acid content and higher sugar content. Moreover, MBC had a fruitier note than the unripe mango and during the process, it developed a good-fermented aroma, which is often described as a wine-like aroma.

In addition to the mass transfer effect, microorganisms also have a possibility of altering the metabolic profiles of MBC. Despite a low pH and high osmotic pressure environment, some microorganisms have survived during the MBC production process. For instance, Sumpavapol et al. (6) reported the presence of lactic acid bacteria (LAB) and yeast strains in MBCs. Furthermore, Maneerat and Meebun (2) reported the viability of bacteria and yeast in commercial MBCs regardless of the low pH range (2.49–3.08). The growth of LAB occurred in brine during the initial fermentation stage of unripe mango, which was later replaced by fermentative yeast in pickled mango (7). In addition, a study by Chen et al. (8) reported that *yan-taozih* (pickled peaches) discovered the importance of microbial diversity in the product’s organoleptic characteristics. It revealed that an increase in lactic acid concentration, a fermentative metabolite, resulted in a higher sensory evaluation score. These studies suggest that metabolites derived from microorganisms could influence the organoleptic characteristics of fermented products. It should be noted that MBC, in general, is not considered a fermented product; however, it was hypothesized that the mild fermentation that occurred during the production process might influence the overall quality. Moreover, Indrati et al. (4) observed fermentative metabolites in commercial MBCs that affected consumers’ preferences. Some of these metabolites, such as γ-aminobutyric acid (9, 10) and short-chain fatty acids (11), are beneficial for health. Behera et al. (12) and Tamang et al. (13) reviewed the benefits of the microbiome in fermented foods. These microorganisms are able to synthesize various metabolites as mentioned earlier. Moreover, it degrades anti-nutritive compounds, such as cyanogenic glycosides, indigestible oligosaccharides, etc.

The combination of metabolomics with multivariate statistical analysis provides beneficial knowledge about food products due to the ability to detect distinct metabolites or metabolite profiles (14, 15). In general, untargeted metabolomics offers a great opportunity to discover new insight information due to a wide range of metabolites detected (16). For example, untargeted metabolomics can distinguish the dynamic metabolite changes during the processing steps of *doenjang*, a fermented soybean paste (17), and black tea (18). Although the applications of metabolomics have been explored in different foods in various food research fields, there is no study of metabolic changes of MBC throughout the production process. The low pH of unripe mango (from the raw material) and the high salt and high-sugar content of immersing solutions could create a hostile environment. Furthermore, these conditions may alter the microbial community and microbial metabolites, which later affect the product quality. Hence, this study aimed to investigate metabolic profile alteration of MBC during the production process. This study revealed the changes of MBC metabolites during the sequential immersion process, which is beneficial for further product development and process optimization.

## Materials and methods

### Chemicals

Standard compounds of sugar as glycerol, meso-erythritol, xylose, arabinose, ribose, xylitol, arabitol, adonitol, fructose, mannose, glucose, mannitol, myo-inositol, sucrose, maltose, and reagents, i.e., N-methyl-N-(trimethylsilyl)-trifluoroacetamide (MSTFA), trimethylchlorosilane (TMCS), 2-methyl 3-heptanone were purchased from Sigma-Aldrich (St. Louis, MO, United States). Pyridine was obtained from Merck (Darmstadt, Germany), *O*-methylhydroxylamine hydrochloride from TCI (Tokyo, Japan), d27-myristic acid from CIL (Tewksbury, MA, United States), methanol from RCI Labscan (Bangkok, Thailand), fatty acid methyl ester (FAME) mixture was purchased from Agilent Technologies (Bangkok, Thailand), and n-alkanes mixture (C7-C40) from Supelco, Inc. (Bellefonte, PA, United States). Sodium chloride (Carlo Erba, Chaussée du Vexin, Val-de-Reuil, France) and a 120 μm DVB/CWR/PDMS SPME Arrow (CTC Analytics AG, Zwingen, Switzerland) were utilized for volatile extraction.

### Ma-Muang Bao Chae-Im sampling and production process

**FIGURE 1:**
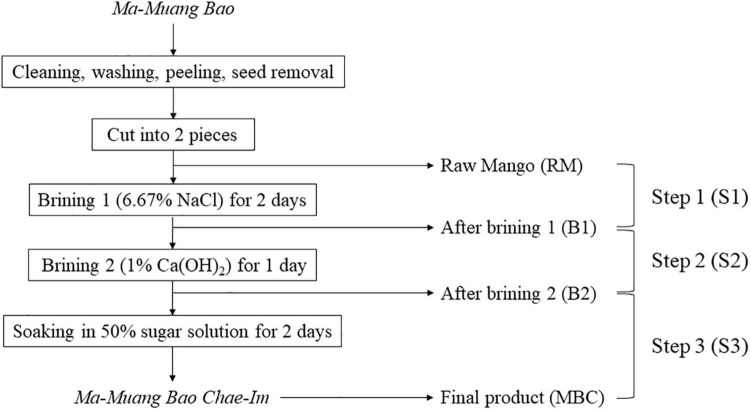
MBC production process and sample collections.

Raw mango (*Ma-Muang Bao*) and mango flesh from each production step were purchased in May 2019 from the Prompsub kaset limited partnership in Hat Yai, Songkhla Province, Thailand. In the first brining stage, 13 kg of raw mangoes were immersed in 6.67% (w/v) NaCl brine (30l) using a 50l plastic bucket with a lid for 2 days. Then, it was turned over after 24 h, so the upper and lower layer of mangoes switched. The next immersion (brining 2) was using 1% Ca(OH)_2_ and the same ratio of mangoes to brine as the previous immersion. This immersion step was done for 1 day. The last immersion step was employing 50% sugar solution for 2 days. Mangoes from the prior stage were transferred to a 60l plastic bucket with a lid with a ratio of 3:2 to mangoes to the sugar solution. Similar to brining 1, the mangoes were overturned after 24h. Raw mangoes (RM) were collected before the first brining process. While the rest of the sample collection was done during the production process, which consisted of mangoes after the first brining (B1), after the second brining (B2), and the final product (MBC). All samples were collected randomly (Figure 1). It was immediately transported to the cold storage facility of the Faculty of Agro-Industry, Prince of Songkla University, within 15 min using a Styrofoam cooler. Each collected sample was ground using an electrical blender (Sharp, Thailand) and then kept at −40°C until further analysis.

### Volatile metabolite analysis by headspace solid-phase micro-extraction (HS-SPME) GC/Q-TOF

Sample preparation of volatile analysis was carried out using a method described by Aprea et al. (19) with modification. All samples (RM, B1, B2, and MBC) were thawed at room temperature (25°C) before the analysis. A 20 ml amber glass vial equipped with a PTFE/silicone septum screw-cap was used in this analysis. Sample (2.5 g), 2.5 ml of deionized water, 1 g of sodium chloride, and 20 μl of 2-methyl 3-heptanone (as an internal standard; IS) were added to the vial. The Agilent 7890B-7250 GC/Q-TOF equipped with an autosampler RTC PAL3 system and DB-Wax capillary column (30 m × 0.25 mm, 0.25 μm, Agilent Technologies, United States) were employed for metabolomics analysis. The sample was equilibrated for 10 min at 60°C and then extracted using SPME DVB/CWR/PDMS Arrow for 60 min at 60°C. The HS-SPME Arrow extract was desorbed from the fiber at 250°C for 2 min. The injection port was operated in a split ratio of 1:10, and 99.999% pure helium was used as the carrier gas at a constant flow rate of 1 ml/min. The initial oven temperature program was set at 45°C for 2.5 min, then raised to 250°C at a rate of 10°C/min, and held for 2 min. The transfer line and the electron ionization source (70 eV) temperature were maintained at 290 and 240°C, respectively. The data were collected at 50 Hz intervals over the *m/z* range of 20–350 amu.

### Non-volatile metabolite analysis by GC/Q-TOF

A homogenized sample (2 g) was extracted using 80% methanol (5 ml) including myristic acid isotope (D27) (25 μl of 50 ppm) as an internal standard (IS). The mixture was sonicated for 30 min at room temperature, then centrifuged for 10 min at 12,857 × *g* at 4°C (20). The supernatant (25 μl) was dried using a vacuum concentrator (Eppendorf concentrator plus, Germany) for 30 min at 60°C. Derivatization of metabolites was prepared using a modified method described by Fiehn (21). First, derivatization was utilized by a 50 μl *O*-methylhydroxylamine hydrochloride (40 mg/ml), and the second 50 μl MSTFA (+ 1% TMCS). The mixture was incubated at 35°C for 90 min for the first and at 38°C for 30 min for the second derivatization. The derivatized sample was analyzed using an Agilent 7890B gas chromatography equipped with an Agilent 7250 GC/Q-TOF. The injection temperature was operated at 250°C in a split ratio of 1:10, and helium (99.999%) was used as the carrier gas at a constant flow rate of 1 mL/min. The chromatographic separation was performed on a DB-5MS DG capillary column (30 m × 0.25 mm, 0.25 μm, Agilent Technologies, United States). The initial oven temperature program was set at 60°C for 1 min, then raised to 325°C at a rate of 10°C/min, and held for 10 min. The transfer line and electron ionization (70 eV) source temperature were maintained at 290 and 250°C, respectively. The data were collected at 5 Hz intervals over the *m/z* range of 50–600 amu.

### Data processing and analysis

All raw data files were transformed to Agilent SureMass format using the MassHunter Unknown Analysis ver. 10.0 software (Agilent, United States) for data treatment. The minimum SNR (signal-to-noise ratio) threshold was set at 10, absolute peak area 250,000 counts, and match factor 70 was implemented as a cut-off to detect and deconvolute the peak. The National Institute of Standards and Technology (NIST14) library was employed to attain peak annotation for volatile metabolites, while the identification of non-volatile metabolites was performed based on NIST14, Fiehn GC/MS Metabolomics RTL Library (Agilent, United States), and our in-house database. The validation of metabolite identification was accomplished by comparing the linear retention index (LRI) from the library with the one that was calculated with a homologous series of n-alkanes (C7–C40) for volatile and a mixture of FAME marker, n-alkanes (C7–C40) and authentic standards (whenever possible) for non-volatile, which were determined under identical conditions as in the samples. Peak area normalization was done by calculating the compound peak area to IS to reduce variation among batches (22).

### Titratable acidity and pH analyses

Titratable acidity was utilized through a direct titration of 0.1 N sodium hydroxide with phenolphthalein as the indicator, which was expressed in grams of citric acid per 100 g sample (23). While digital pH meter (Mettler-Toledo AG, Switzerland) was employed to measure the pH in the sample. Distilled water (100 ml) was added to an Erlenmeyer flask containing a 10 g homogenized sample then the mixture was stirred for 1 min and pH was measured after 3 min rest (23).

### Statistical analysis

Descriptive statistics, one-way ANOVA, Tukey HSD *post hoc* test of pH and titratable acidity, and independent *t*-test for discriminant markers were done using SPSS 17 (IBM, New York, NY, United States). Principal component analysis (PCA) and orthogonal partial least square-discriminant analysis (OPLS-DA) were performed with Pareto scaling performed in the SIMCA software Version 16.0.2 (Umetrics, Umeå, Sweden). To quantify metabolites changes in each immersion step and obtain the correlation among discriminant metabolites, fold change [log2 (FC)] and Pearson correlation analysis, respectively, (MetaboAnalyst 5.0) were applied without data normalization. Titratable acidity (TA) and pH data were obtained in three biological replicates, while volatile and non-volatile metabolites analyses were performed in three technical and biological replicates.

## Results

### Overview of volatile and non-volatile metabolites during the production process of Ma-Muang Bao Chae-Im

**FIGURE 2:**
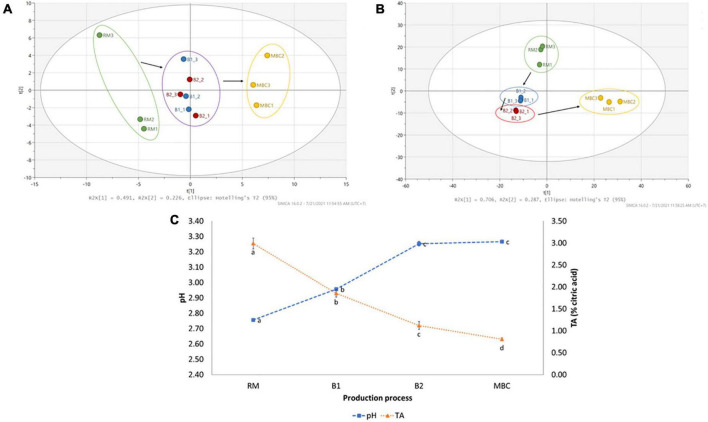
PCA score plot of volatile **(A)** and non-volatile **(B)** analyses of mango flesh (RM: raw mango, B1: after brining 1, B2: after brining 2, MBC: final product) during the production process. Changes in pH and titratable acidity (TA) of mango flesh during immersion process **(C)**. Data of pH and TA are given as mean ± SD (*n* = 3); different letter indicates a significant difference (Tukey HSD, *p* < 0.05).

The changes in various volatile and non-volatile metabolites were observed during the production process. It was noted that 82 volatile and 40 non-volatile metabolites were identified from the untargeted profiling approach. All volatile metabolites were identified based on the NIST14 library, whereas non-volatile metabolites employed various resources (Supplementary Tables 1, 2). The log2(FC) was conducted to quantify metabolite changes in each production step (Supplementary Tables 3, 4). Figure 2 shows a clear separation of mangoes in each processing step using volatile (Figure 2A) and non-volatile (Figure 2B) metabolites, as illustrated by PCA score plots. Although brining processes did not give a clear distinction, RM and MBC were in the opposite area separated by principal component 1 (PC1), whereas non-volatile by both principal components (PC1 and PC2). In addition, an increase in pH level of mango flesh from 2.76 (RM) to 3.27 (MBC) and a decrease in titratable acidity (TA) of mango flesh were observed (Figure 2C).

### Changes in Ma-Muang Bao Chae-Im volatile metabolite profiles during the immersion processes

**FIGURE 3:**
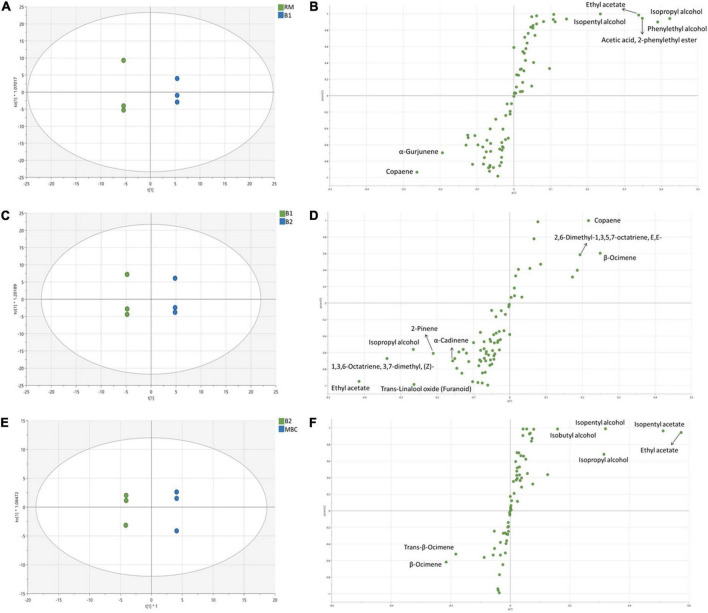
OPLS-DA score plot and S-plot of volatile metabolites of S1 **(A,B)**, S2 **(C,D)**, and S3 **(E,F)**. Each dot in the score plot represents a biological replicate.

Figures 3A–D shows the OPLS-DA score plot and S-plot, which gave a clearer separation between production stages as compared with the PCA method and with identification of potential discriminant markers with cut-off p(corr) ≤ 0.5. During the S1 step (Figures 3A,B), the score plot and S-plot obtained from RM and B1 showed clear discrimination, and the model has R2X = 0.917, R2Y = 0.97, and Q2 = 0.914. Copaene and α-Gurjunene were the discriminant markers of RM, while B1 was isopropyl alcohol; phenylethyl alcohol; isopentyl alcohol; ethyl acetate and acetic acid; and 2-phenylethyl ester. Figures 3C,D shows the score and S-plot of the OPLS-DA model of the S2 stage (B1 and B2). The OPLS-DA model has R2X = 0.849, R2Y = 0.995, and *Q2* = 0.894, indicating a good quality of the model, showing its ability to distinguish between B1 and B2. Ethyl acetate, isopropyl alcohol, *trans*-linalool oxide, 2-Pinene, 1,3,6-Octatriene, 3,7-dimethyl (Z), and α-Cadinene were discriminant markers of B1, while copaene, β-Ocimene, and 2,6-Dimethyl-1,3,5,7-octatriene, E,E- were discriminant markers of B2. The last immersion process of MBC was S3 (Figures 3E,F). The OPLS-DA model has R2X = 0.772, R2Y = 0.995, and *Q2* = 0.971. The discriminant markers of B2 were β-Ocimene and *trans*-β-Ocimene, while MBC were fermentative metabolites comprised of ethyl acetate, isopentyl acetate, isopentyl alcohol, isopropyl alcohol, and isobutyl alcohol.

### Changes in Ma-Muang Bao Chae-Im non-volatile metabolite profiles during the immersion processes

**FIGURE 4:**
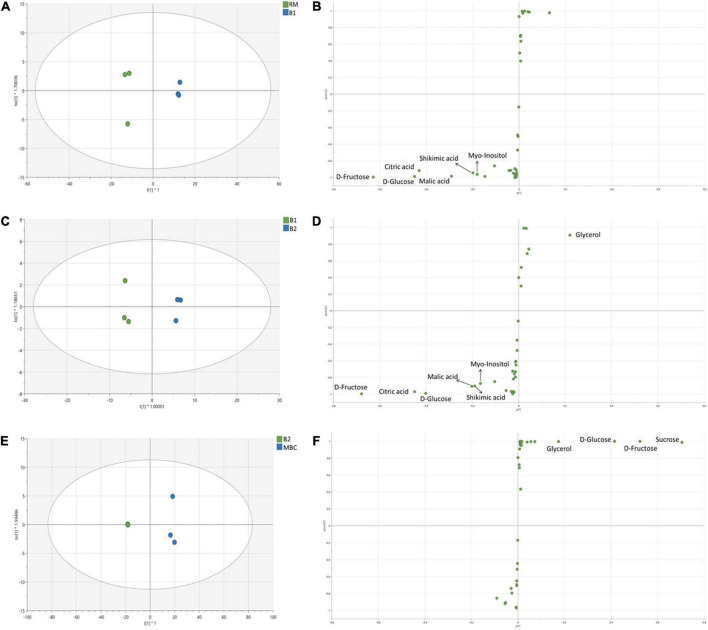
OPLS-DA score plot and S-plot of non-volatile metabolites of S1 **(A,B)**, S2 **(C,D)**, and S3 **(E,F)**. Each dot in the score plot represents a biological replicate.

In the first immersion process (S1), both RM and B1 were clearly distinguished (Figure 4A). OPLS-DA model of S1 has R2X = 0.998, R2Y = 0.998, and *Q2* = 0.994. In this step, six discriminant markers, i.e., D-fructose, D-glucose, citric acid, malic acid, shikimic acid, and myo-inositol, were identified, which belong to RM (Figure 4B). The OPLS-DA model of S2 stage has R2X = 0.984, R2Y = 0.995, *Q2* = 0.989, and similar discriminant markers with S1 (Figures 4C,D). Sugars and organic acids were correlated with B1, while glycerol with B2. In the next stage (S3 step), OPLS-DA has R2X = 0.997, R2Y = 0.997, *Q2* = 0.992, and all discriminant markers were associated with MBC, i.e., sucrose, D-fructose, D-glucose, and glycerol (Figures 4E,F).

### Metabolites alteration and correlation during each immersion process

**FIGURE 5:**
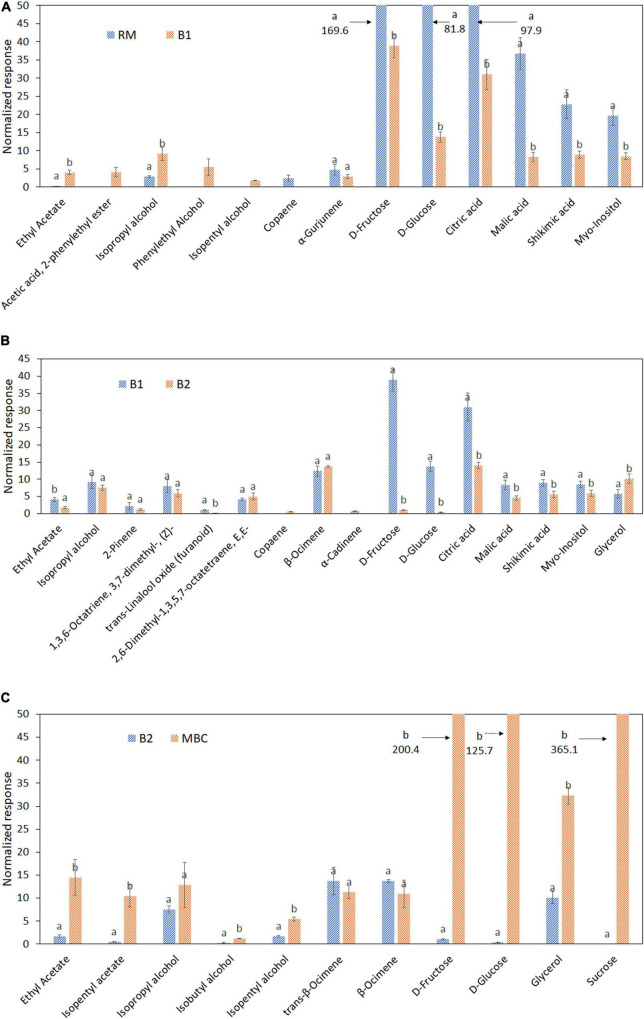
Discriminant metabolites of raw mango (RM), after brining 1 (B1), after brining 2 (B2), and MBC (final product) in each immersion process: S1 **(A)**, S2 **(B)**, and S3 **(C)**. Different letter indicates a significant difference (independent *t*-test, α = 0.05). Mean value ± standard deviation was used (*n* = 3).

**FIGURE 6:**
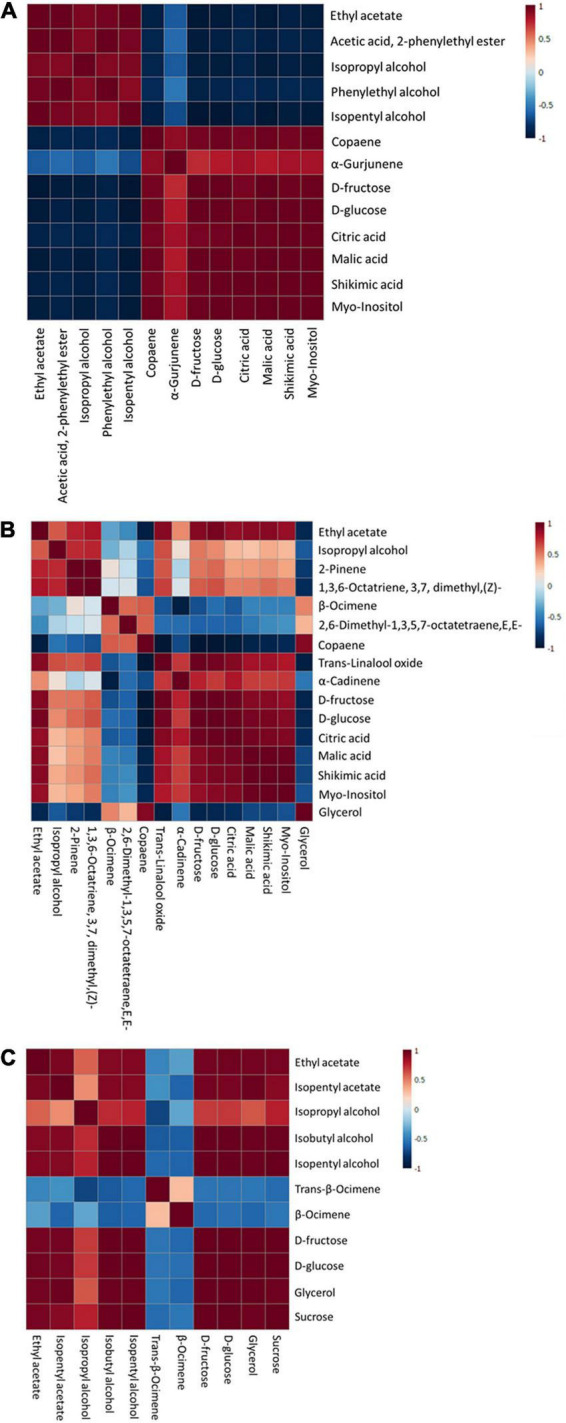
Pearson correlation analysis of discriminant markers of each immersion process: S1 **(A)**, S2 **(B)**, and S3 **(C)**.

The alteration of selected discriminant markers in each step is visualized in the bar graphs and heatmaps based on Pearson correlation to investigate the discriminant markers relationship (Figures 5, 6). Thirteen metabolites were distinguished as discriminant markers in the S1 step, i.e., ethyl acetate; acetic acid, 2-phenylethyl ester; isopropyl alcohol; phenylethyl alcohol; isopentyl alcohol; copaene; α-Gurjunene, D-fructose; D-glucose; citric acid; malic acid; shikimic acid; and myo-inositol (Figure 5A). In the S2 step, 16 discriminant markers were observed, containing ethyl acetate; isopropyl alcohol; 2-Pinene; 1,3,6-Octatriene, 3,7-dimethyl, (Z)-; 2,6-Dimethyl-1,3,5,7-octatriene, E,E-; copaene; *trans*-linalool oxide; α-Cadinene; β-Ocimene; D-fructose; D-glucose; citric acid; malic acid; shikimic acid; myo-inositol; and glycerol (Figure 5B). Figure 5C shows a total of 11 discriminant markers in the S3 step, consisting of ethyl acetate, isopentyl acetate, isopropyl alcohol, isobutyl alcohol, isopentyl alcohol, *trans*-β-Ocimene, β-Ocimene, D-fructose, D-glucose, glycerol, and sucrose.

The relationship of discriminant markers is evaluated by the Pearson correlation method during each immersion process (Figure 6). During the first immersion process (S1), esters and alcohols have a negative correlation with copaene, α-Gurjunene, sugar, and non-volatile organic acids (Figure 6A). An interesting result was found in the S2 step, where copaene; 2,6-dimethyl-1,3,5,7-octatetraene, E,E-; and β-Ocimene has a positive correlation with glycerol, whereas negatively correlated with D-glucose and D-fructose (Figure 6B). In the next stage (S3), a positive correlation was found among fructose, glucose, sucrose, and fermentative metabolites, i.e., esters, alcohols, and glycerol (Figure 6C).

## Discussion

Ma-Muang Bao Chae-Im is an osmotically dehydrated mango that has a unique flavor. During the osmotic dehydration process of MBC, mass transfer between the mango flesh and osmotic solutions happened, triggering the alteration of volatile and non-volatile compounds of mango. These compounds that originated from mango and developed during the immersion processes play important role in the flavor formation of MBC, which is projected in the separation of mango fleshes in PCA score plots. Even though mango flesh in B1 and B2 processes close to each other, they were thoroughly distinguished from RM and MBC. This event suggested the similarity between metabolite profiles of mango after bringing processes and the differences between unripe mango, mango after brining processes, and after immersion in sugar solution. Sugars and organic acid content in mango fluctuated throughout the production process. Both metabolite classes were observed in a lower amount in B1 and B2 compared to RM. Although the sugar content of MBC surged after immersion in hypertonic sugar solution due to sugar impregnation, the organic acid content shows a declining tendency. These organic acids, especially non-volatile organic acids, impart tartness to unripe mango (4, 24). Several organic acids originated from the unripe mango, with citric acid and malic acid as the main source of mango acidity. As a consequence of lower organic acid content, MBC has less tartness than RM. This occurrence is in line with the increasing trend of pH and declining TA throughout the MBC production process. In the volatile profile, terpenes and terpenoids as the main volatile components in unripe mango have a green and fruity aroma (25). These mango metabolites were present through the immersion processes.

Overall, our data suggested that changes in volatile and non-volatile metabolite profiles were not only from the mass transferring between the mango flesh and osmotic solutions but also from microbial activities. It was postulated that the acidic nature of RM might create a hostile environment for microorganisms. In addition, the high osmotic pressure created by high salt or sugar concentrations in an osmotic solution could hinder microbial growth (26). However, some yeasts and bacteria might be able to survive regardless of the acidic and hypertonic conditions. Yu et al. (27) observed the growth of bacteria, yeast, and fungi in osmotically dehydrated blueberries. In addition, Azeez et al. (28) found viable fungi in osmotically dehydrated tomato slices. These indigenous microbiotas, which are usually found on the fruit surface, become the main initiator of spontaneous fermentation because of their adaptive nature to the mango matrix (29). A study in by (30) showed that *Leuconostoc mesenteroides*, *Pediococcus pentosaceus*, and *Lactobacillus brevis* were identified as native LAB from mango. While some yeast, *viz*. *Hanseniaspora, Pichia, Candida*, and *Saccharomyces* were isolated from mango pulp (31). Most of these native microorganisms were found (12) and were able to synthesize ester and alcohol in traditional pickles (32), which are found in abundance in MBC.

One key reason for those organisms to withstand the high osmotic pressure and acidic conditions is their ability to produce some metabolites to maintain intracellular pH homeostasis. The first strategy of microorganisms to encounter the low pH condition is proton consumption. Decarboxylation reaction is often employed for this purpose. Second, basic metabolites are synthesized to counteract the acidic condition, and then the last strategy is proton elimination by releasing it at the expense of ATP consumption (33). Alcohol and ester classes were produced greatly throughout the MBC production process. Both products were common metabolites produced by yeast. Higher alcohol (alcohol that has more than two carbon atoms) was produced through the Ehrlich pathway, which comprises transamination, decarboxylation, and reduction of branched-chain amino acid. Moreover, ester was synthesized from acyl-CoA and its respective alcohol (34). During fermentation, acetyl Co-A and medium-chain fatty acid (MCFA), an intermediate product of fatty acid synthesis, were accumulated in the cell. To avoid the production of free acetic and accumulation of MCFA, acetate esters, and ethyl esters were produced. The diffusion of esters through the plasma membrane into the environment resulted in a decreasing intracellular toxic compound accumulation (35). Knoll et al. (36) observed a rise in esters concentration in white wines during malolactic fermentation in an acidic condition. Furthermore, γ-aminobutyric acid (GABA), a derivation of glutamate through a proton consumption system named glutamate decarboxylase, was synthesized to regulate the internal pH (37). Laroute et al. (38) revealed that GABA biosynthesis only occurred when *Lactococcus lactis* was exposed to an acidic environment and was affected by the presence of chloride ions. Therefore, GABA production was enhanced greatly during the first brining process and then declined after the second bringing. However, only a trace amount of glutamate was present in mango (24). Therefore, it is suggested that L-5-oxoproline in RM was metabolized into glutamate via ATP-dependent 5-oxoprolinase by yeast (39).

Most esters impart fruity and floral notes to the product, while GABA has a bitter taste. Furthermore, acetic acid was found in mango after immersion processes. This volatile acid was produced by yeast through pyruvate metabolism and lactic acid bacteria (LAB) via pyruvate metabolisms and citric acid conversion (40). Thus, microbial metabolites could affect the volatile and non-volatile compound profiles and alter mango flesh’s aroma and flavor of the final product.

Orthogonal partial least square-discriminant analysis as a supervised method (41) was employed to improve the discrimination power for discovering the potential discriminant markers for immersion processes. The OPLS-DA can reveal systematic trends within the data, which incline toward the objectives. Moreover, S-plot (42) was also employed to identify potentially significant metabolites based on their reliability and contributions to the OPLS-DA model. The S-plot *x*-axis (p[1]) describes the magnitude of each variable in X, while the *y*-axis (p(corr)[1]) the reliability of each variable in X.

Copaene; α-Gurjunene; isopropyl alcohol; phenylethyl alcohol; isopentyl alcohol; ethyl acetate; and acetic acid, 2-phenylethyl ester were discriminant markers of volatile metabolite in S1. As aforementioned, ester and higher alcohol were synthesized as the common yeast metabolites, while copaene and α-Gurjunene were found in unripe mango (43). In addition, D-glucose, D-fructose, and sucrose, along with citric, malic, and shikimic acid, were major metabolites in various mango cultivars (24) and were found as non-volatile discriminant markers. During the first brining process (S1), some solutes from the mango flesh migrated into the brine solution and *vice versa*, salt from the brine into mango flesh. As a result, lower amounts of copaene, α-Gurjunene, D-fructose, D-glucose, citric acid, malic acid, shikimic acid, and myo-inositol were observed in B1 compared to RM. Furthermore, the drastic decrease in monosaccharides indicated that some microorganisms could survive in this stage and utilize it as a carbon source. Studies about natural fermented pickled products using brine reported viable microorganisms during the immersion and final products. Pérez-Díaz et al. (44) observed a number of native bacteria during brining process of pickled cucumber. While several wild yeasts were found in the natural fermentation process of eggplant in brine (45). However, only certain microorganisms are able to assimilate fructose. Microorganisms that have a preference to use fructose as a carbon source than glucose are called fructophilic microorganisms. The depletion of fructose during S1 indicates that fructophilic microorganisms were viable. *Hanseniaspora guilliermondii* showed a fructophilic behavior in medium with high sugar concentration (46).

Ethyl acetate; isopropyl alcohol; *trans*-linalool oxide; 2-Pinene; 1,3,6-Octatriene, 3,7-dimethyl, (Z)-; α-Cadinene; copaene; β-Ocimene; and 2,6-Dimethyl-1,3,5,7-octatriene, E,E- were the discriminant markers of volatile metabolites in S2, while D-fructose, D-glucose, citric acid, malic acid, shikimic acid, and myo-inositol were from non-volatile metabolites. Most discriminant marker levels were decreasing in S2 due to the mass transfer between mango flesh and calcium hydroxide solution, although higher levels of copaene and glycerol were observed. This event suggested an occurrence of the hydrolysis process of mango glycosides by yeast glucosidases enzyme. This process cleaves the glycosidic bond of terpene glycosides and then releases volatile aglycone and sugar moiety (47).

Volatile discriminant markers found in S3 were β-Ocimene, *trans*-β-Ocimene, ethyl acetate, isopentyl acetate, isopentyl alcohol, isopropyl alcohol, and isobutyl alcohol. Moreover, sucrose, D-fructose, D-glucose, and glycerol were the non-volatile discriminant markers. The dominance of fermenting yeasts in S3 might promote the production of ethyl acetate, isopentyl acetate, isopropyl alcohol, isobutyl alcohol, isopentyl alcohol, and glycerol as the fermentative metabolites. Glycerol is a by-product of alcoholic fermentation by yeasts to maintain redox balance and has a role as an osmolyte in yeasts when exposed to a harsh environment such as high osmotic pressure and low pH (48, 49). It was observed that the spike of fructose, glucose, and sucrose content in MBC suggested sugar impregnation during the S3 step.

The negative correlation of esters and alcohols with copaene, α-Gurjunene, sugar, and non-volatile organic acids indicated sugar consumption by microorganisms; hence, ester and alcohol synthesis occurred during the S1 step. An interesting result was found in the S2 step, where copaene; 2,6-dimethyl-1,3,5,7-octatetraene, E,E-; and β-Ocimene have a positive correlation with glycerol, whereas negatively correlated with D-glucose and D-fructose. As mentioned earlier, this suggested a possibility of yeast’ glucosidase enzyme liberation that can hydrolyze terpene glycoside. This enzyme is inhibited by a high concentration of glucose, fructose, sucrose, and ethanol, depending on the organisms. A study in 2011 by Swangkeaw and colleagues observed that β-glucosidase from *Hanseniaspora* sp. was inhibited by ethanol and high glucose concentration but not fructose and sucrose. On the other hand, β-glucosidase from *Pichia anomala* was inhibited by glucose, fructose, and sucrose but not ethanol (50). Therefore, due to the low inhibitor agents, β-glucosidase was active during the S2 step. In the next stage (S3), a positive correlation was found between fructose, glucose, sucrose, and fermentative metabolites, i.e., esters, alcohols, and glycerol. It indicated that a rise of fermentative metabolites was induced by the abundance of sugar levels in MBC. The significant increase in ester and alcohol groups during the production process suggested the viability and dominance of yeast in the MBC production process.

## Conclusion

The investigation of the alteration of metabolic profiles of MBC resulted in 82 volatile and 41 non-volatile metabolites during the MBC production process through untargeted metabolomics profiling by GC/Q-TOF. Through OPLS-DA, discriminant markers of each immersion process were obtained. These discriminant markers were identified as alcohols, esters, terpenes, furans, non-volatile organic acids, monosaccharides, sugar alcohols, and disaccharides that fluctuated throughout the process. Mass transfer between mango flesh and osmotic solution caused the decline of terpenes, terpenoids, organic acids, and sugars that originated from RM. On the other hand, some metabolites were formed as an environmental stress response from microorganisms during the MBC production process. The elevation of fermentative metabolites, i.e., esters, higher alcohols, and glycerol levels, might be relevant to yeast domination during the process. Further investigation will be required to elucidate the microbial alteration during the MBC production process thus can bring a deeper understanding of flavor development and metabolites production mechanisms. Moreover, the presence of some beneficial health metabolites like GABA during the production process could be enhanced to create more value-added products. The acquired knowledge from this study would be beneficial to obtain a good quality of product development and optimization.

## Data availability statement

The original contributions presented in this study are included in the article/Supplementary material, further inquiries can be directed to the corresponding authors.

## Author contributions

NI participated in conceptualization, conducted the experiment, generated the data, and wrote the original draft. PS and AP partook in conceptualization, methodology, reviewing, editing, supervising, and accommodated this work. SK contributed to conceptualization, methodology, validation, reviewing, editing, and accommodated this study. NP and PP took a part in reviewing the draft and polishing the grammar. All authors contributed to the article and approved the manuscript.
