# Supplementary material for: Metabolic profiles alteration of Southern Thailand traditional sweet pickled mango during the production process
Source: Front Nutr. 2022 Sep 8;9:934842. doi: 10.3389/fnut.2022.934842 (PMC9493497; doi:10.3389/fnut.2022.934842)
Supplement: Supplementary file 1 [file Data_Sheet_1.docx]

**Supplementary Material**

**Supplementary Table 1.** Volatile metabolites of raw mango (RM), after brining1 (B1), after brining2 (B2), and final product (MBC).

| RT | Compound Name | RI Lib | RI Cal^a^ | RM | B1 | B2 | MBC |
| --- | --- | --- | --- | --- | --- | --- | --- |
| 3.4168 | Ethyl Acetate | 888 | 892 | 0.263±0.031 | 4.067±0.608 | 1.716±0.323 | 14.496±3.864 |
| 6.7003 | Isopentyl acetate | 1122 | 1118 | - | 0.385±0.037 | 0.459±0.036 | 10.476±2.414 |
| 16.104 | Acetic acid, 2-phenylethyl ester | 1813 | 1828 | - | 4.191±1.277 | 5.318±1.863 | 6.654±1.497 |
| 16.3226 | Dodecanoic acid, ethyl ester | 1841 | 1845 | - | - | 0.080±0.013 | 0.308±0.034 |
| 18.3055 | Isopropyl myristate | 2027 | 2042 | 0.064±0.065 | 0.069±0.085 | 0.054±0.03 | 0.124±0.061 |
| 18.4316 | Tetradecanoic acid, ethyl ester | 2049 | 2055 | - | 0.079±0.019 | 0.090±0.018 | 0.111±0.016 |
| 20.0261 | Hexadecanoic acid, ethyl ester | 2251 | 2224 | - | - | - | 0.069±0.01 |
| 20.6171 | Ethyl 9-hexadecenoate | 2281 | 2290 | - | - | - | 0.112±0.016 |
|  | **Total ester** |  |  | **0.327±0.042^a^** | **8.791±1.825^a^** | **7.717±2.2^a^** | **32.348±6.013^b^** |
| 3.9995 | Isopropyl alcohol | 927 | 936 | 2.959±0.257 | 9.149±1.903 | 7.528±0.801 | 12.857±4.915 |
| 6.4502 | Isobutyl alcohol | 1092 | 1102 | 0.089±0.154 | 0.166±0.144 | 0.261±0.105 | 1.198±0.075 |
| 8.2296 | Isopentyl alcohol | 1209 | 1209 | - | 1.795±0.054 | 1.657±0.218 | 5.435±0.415 |
| 17.1159 | Phenylethyl Alcohol | 1906 | 1924 | - | 5.514±2.318 | 5.715±0.794 | 6.357±1.431 |
|  | **Total alcohol** |  |  | **3.048±0.339^a^** | **16.624±3.664^b^** | **15.161±1.06^b^** | **25.847±5.54^c^** |
| 4.5563 | Pentanal | 979 | 978 | 0.065±0.027 | 0.034±0.033 | 0.032±0.028 | 0.029±0.028 |
| 6.1076 | Hexanal | 1083 | 1080 | 0.049±0.047 | 0.114±0.011 | 0.043±0.038 | 0.204±0.041 |
| 9.3581 | Octanal | 1289 | 1289 | 0.121±0.073 | 0.103±0.031 | 0.061±0.012 | 0.072±0.022 |
| 9.9268 | 2-Heptenal, (E)- | 1323 | 1329 | 0.158±0.031 | 0.301±0.11 | 0.234±0.084 | 0.317±0.121 |
| 10.875 | Nonanal | 1391 | 1396 | 0.113±0.039 | 0.098±0.043 | 0.065±0.064 | 0.062±0.054 |
| 11.4115 | 2-Octenal, (E)- | 1429 | 1436 | 0.134±0.022 | 0.238±0.057 | 0.110±0.102 | 0.111±0.1 |
| 14.1322 | 2-Decenal, (E)- | 1644 | 1633 | 0.238±0.054 | 0.278±0.085 | 0.154±0.035 | 0.191±0.047 |
|  | **Total aldehyde** |  |  | **0.878±0.12^a^** | **1.166±0.28^a^** | **0.698±0.316^a^** | **0.986±0.309^a^** |
| 5.2105 | 2-Pinene | 1028 | 1023 | 2.936±0.537 | 2.183±1.04 | 1.238±0.242 | 1.010±0.301 |
| 7.2388 | β-Myrcene | 1161 | 1152 | 1.535±0.197 | 1.269±0.146 | 0.921±0.181 | 0.816±0.13 |
| 7.5347 | (+)-4-Carene | 1149 | 1170 | 0.062±0.011 | - | - | - |
| 7.8484 | L-Limonene | 1199 | 1190 | 0.531±0.146 | 0.265±0.043 | 0.184±0.058 | 0.105±0.091 |
| 7.9971 | α-Phellandrene | 1167 | 1201 | 0.160±0.085 | - | - | - |
| 8.4113 | 1,3,6-Octatriene, 3,7-dimethyl-, (Z)- | 1235 | 1227 | 7.872±3.211 | 8.079±1.861 | 5.870±1.011 | 5.444±1.045 |
| 8.6639 | β-Ocimene | 1250 | 1243 | 11.426±1.723 | 12.349±1.489 | 13.676±0.294 | 10.911±3.015 |
| 8.9156 | trans-β-Ocimene | 1250 | 1259 | 11.837±4.6 | 12.484±1.089 | 13.704±2.981 | 11.344±1.505 |
| 9.2396 | γ-Terpinene | 1246 | 1282 | 0.349±0.247 | 0.126±0.103 | 0.035±0.036 | - |
| RT | Compound Name | RI Lib | RI Cal^a^ | RM | B1 | B2 | MBC |
| 10.5377 | 2,4,6-Octatriene, 2,6-dimethyl-, (E,E)- | 1379 | 1372 | 0.204±0.079 | 0.219±0.093 | 0.170±0.057 | 0.151±0.007 |
| 10.8584 | 2,4,6-Octatriene, 2,6-dimethyl- | 1375 | 1395 | 1.147±0.783 | 1.041±0.455 | 0.662±0.17 | 0.586±0.157 |
| 11.3994 | p-Mentha-1,5,8-triene | 1408 | 1435 | 1.243±0.324 | 1.472±0.308 | 1.170±0.149 | 1.300±0.214 |
| 11.5752 | 2,6-Dimethyl-1,3,5,7-octatetraene, E,E- | 1460 | 1449 | 5.217±1.646 | 4.154±0.301 | 4.982±0.947 | 5.165±0.976 |
| 14.4328 | trans-Ocimenol | 1650 | 1648 | 0.822±0.72 | 0.850±0.406 | 0.410±0.116 | 0.368±0.059 |
| 14.8779 | trans-Pinocarveol | 1664 | 1671 | 0.123±0.02 | 0.577±0.186 | 0.602±0.171 | 0.713±0.174 |
| 15.3869 | Neryl Acetate | 1724 | 1697 | 0.020±0.03 | 0.123±0.04 | 0.093±0.026 | 0.137±0.029 |
| 16.0354 | trans-2-Caren-4-ol | 1816 | 1823 | 0.094±0.015 | 0.135±0.031 | 0.099±0.024 | 0.172±0.016 |
| 16.2213 | β-Damascenone | 1823 | 1839 | 0.121±0.042 | 0.077±0.017 | - | 0.026±0.023 |
| 16.4846 | Geranylacetone | 1859 | 1864 | 0.128±0.027 | 0.270±0.061 | 0.201±0.041 | 0.138±0.02 |
|  | **Total monoterpene & terpenoid** |  |  | **46.046±14.23^a^** | **45.777±6.98^a^** | **44.016±3.58^a^** | **38.387±6.01^a^** |
| 9.5616 | 1-Octen-3-one | 1300 | 1303 | 0.073±0.034 | 0.138±0.057 | 0.088±0.022 | 0.127±0.05 |
| 10.0926 | 5-Hepten-2-one, 6-methyl- | 1338 | 1341 | - | 0.127±0.025 | 0.107±0.025 | 0.086±0.018 |
| 13.5301 | 6-Methyl-3,5-heptadiene-2-one | 1602 | 1602 | 0.116±0.042 | 0.517±0.13 | 0.253±0.056 | 0.477±0.092 |
|  | **Total ketone** |  |  | **0.189±0.022 ^a^** | **0.782±0.163^b^** | **0.448±0.085 ^ac^** | **0.690±0.112^bc^** |
| 11.7792 | α-Cubebene | 1463 | 1464 | 0.059±0.025 | 0.141±0.039 | - | - |
| 12.1515 | Copaene | 1492 | 1492 | 2.422±0.808 | - | 0.611±0.037 | 0.829±0.116 |
| 12.7966 | α-Gurjunene | 1528 | 1544 | 4.724±1.479 | 2.962±0.547 | 2.886±0.451 | 2.382±0.456 |
| 12.9522 | (-)-Aristolene | 1572 | 1556 | 0.202±0.056 | 0.048±0.005 | - | - |
| 13.0124 | cis-α-Bergamotene | 1562 | 1561 | 0.093±0.047 | 0.129±0.015 | 0.081±0.074 | 0.123±0.013 |
| 13.3596 | β-Ylangene | 1589 | 1589 | 0.068±0.059 | 0.070±0.018 | 0.033±0.029 | 0.031±0.029 |
| 13.4965 | β-Elemene | 1591 | 1600 | 0.049±0.053 | - | - | - |
| 13.5862 | β-Copaene | 1586 | 1604 | 0.140±0.024 | 0.094±0.015 | 0.042±0.037 | 0.048±0.01 |
| 13.6634 | Caryophyllene | 1595 | 1608 | 1.483±0.564 | 0.969±0.185 | 0.736±0.077 | 0.847±0.076 |
| 13.7137 | β-Gurjunene | 1605 | 1611 | 0.175±0.078 | 0.161±0.091 | 0.066±0.013 | 0.066±0.005 |
| 14.2234 | Aromandendrene | 1635 | 1637 | 1.017±0.408 | 0.695±0.139 | 0.578±0.063 | 0.738±0.162 |
| 14.2678 | Alloaromadendrene | 1650 | 1640 | 0.617±0.176 | 0.341±0.11 | 0.264±0.063 | 0.250±0.056 |
| 14.4559 | α-Elemene | 1685 | 1649 | 0.167±0.101 | 0.099±0.087 | 0.097±0.018 | 0.099±0.024 |
| 14.5763 | γ-Selinene | 1682 | 1656 | 1.037±0.368 | 0.664±0.161 | 0.518±0.082 | 0.548±0.09 |
| 14.693 | α-Himachalene | 1661 | 1660 | 3.774±1.349 | 2.797±0.434 | 2.560±0.318 | 2.743±0.425 |
| 14.8203 | Viridflorene | 1697 | 1668 | 0.604±0.271 | 0.117±0.045 | 0.064±0.017 | - |
| 15.0102 | γ-Gurjunene | 1674 | 1678 | 2.568±0.971 | 1.655±0.392 | 1.386±0.168 | 1.492±0.162 |
| 15.1133 | β-Selinene | 1717 | 1683 | 6.486±5.423 | 6.834±2.293 | 6.472±2.662 | 6.513±2.399 |
| 15.3137 | Valencen | 1728 | 1694 | 0.631±0.153 | 0.442±0.089 | 0.353±0.044 | 0.390±0.068 |
| 15.4947 | δ-Cadinene | 1758 | 1716 | 1.262±0.591 | 0.932±0.251 | 0.502±0.439 | 0.771±0.139 |
| RT | Compound Name | RI Lib | RI Cal^a^ | RM | B1 | B2 | MBC |
| 15.504 | γ-Cadinene | 1765 | 1719 | 0.202±0.194 | 0.136±0.124 | 0.334±0.297 | 0.106±0.094 |
| 16.3574 | trans-Calamenene | 1826 | 1852 | 0.082±0.014 | 0.033±0.034 | 0.051±0.007 | - |
| 16.3894 | Germacrene B | 1819 | 1855 | 0.170±0.021 | 0.056±0.05 | 0.067±0.021 | - |
| 17.2633 | β-Calacorene | 1930 | 1938 | 0.070±0.018 | 0.077±0.014 | 0.080±0.035 | 0.072±0.011 |
| 17.2696 | Ledane | 1958 | 1939 | 0.020±0.035 | 0.070±0.061 | 0.068±0.019 | 0.055±0.051 |
| 17.4396 | Palustrol | 1934 | 1956 | 0.078±0.013 | 0.064±0.016 | 0.054±0.018 | 0.035±0.034 |
| 18.7837 | Cubenol | 2080 | 2091 | 0.143±0.072 | 0.174±0.033 | 0.136±0.022 | 0.127±0.016 |
| 19.4407 | Neointermedeol | 2138 | 2161 | 0.180±0.055 | 0.200±0.04 | 0.171±0.029 | 0.156±0.016 |
| 19.8728 | α-Cadinol | 2226 | 2207 | 0.108±0.036 | 0.108±0.017 | 0.089±0.016 | 0.083±0.011 |
|  | **Total sesquiterpene & terpenoid** |  |  | **29.908±13.26 ^a^** | **21.186±5.08 ^a^** | **18.854±4.51 ^a^** | **19.045±3.84 ^a^** |
| 10.9403 | 3-Methyl-2-(2-methyl-2-butenyl)-furan | 1413 | 1401 | 0.148±0.06 | 0.208±0.045 | 0.122±0.015 | 0.299±0.056 |
| 12.0032 | trans-Linalool oxide (furanoid) | 1452 | 1481 | 0.309±0.188 | 1.021±0.131 | 0.110±0.036 | 0.161±0.097 |
|  | **Total furan** |  |  | **0.457±0.245 ^a^** | **1.229±0.161^b^** | **0.232±0.041 ^a^** | **0.460±0.121 ^a^** |
| 11.7214 | Acetic acid | 1449 | 1460 | - | 0.234±0.095 | 0.117±0.038 | 0.184±0.086 |
|  | **Total organic acid** |  |  | **-** | **0.234±0.095 ^a^** | **0.117±0.038 ^a^** | **0.184±0.086 ^a^** |
| 10.4634 | o-Xylene | 1369 | 1367 | 0.413±0.082 | 0.488±0.108 | 0.401±0.111 | 0.405±0.112 |
| 10.9456 | α-Pyronene | 1365 | 1401 | 0.074±0.074 | - | - | - |
| 11.4802 | m-Cymenene | 1440 | 1441 | 0.158±0.078 | - | - | - |
|  | **Total aromatic compound** |  |  | **0.701±0.244 ^a^** | **0.488±0.108 ^a^** | **0.401±0.111 ^a^** | **0.405±0.112 ^a^** |

^a^Linear retention index (LRI) bases on DB-WAX capillary column using a series of alkanes between C_7_-C_40_.

All values are the mean ± SD (n=3) and different letter indicates a significant difference (Tukey HSD, p<0.05).

Metabolites annotation was based on mass spectrum match and RI of each metabolite with NIST14 library.

**Supplementary Table 2.** Non-volatile metabolites of raw mango (RM), after brining1 (B1), after brining2 (B2), and final product (MBC).

| RT | Compound Name | ID^a^ | RI Lib | RI Cal^b^ | RM | B1 | B2 | MBC |
| --- | --- | --- | --- | --- | --- | --- | --- | --- |
| 7.8267 | Lactic Acid (2TMS) | RA | 1066 | 1053 | - | - | 0.083±0.013 | 0.160±0.014 |
| 8.0616 | Glycolic acid (2TMS) | R | 1081 | 1069 | 0.031±0.012 | 0.026±0.023 | 0.016±0.016 | 0.011±0.019 |
| 10.338 | Propanedioic acid (2TMS) | R | 1216 | 1228 | - | - | - | 0.056±0.006 |
| 10.8446 | Phosphoric acid (3TMS) | R | 1286 | 1264 | 0.504±0.174 | 0.064±0.019 | 0.045±0.013 | 0.014±0.025 |
| 11.4147 | Succinic acid (2TMS) | RA | 1321 | 1305 | 0.054±0.003 | 0.546±0.062 | 0.699±0.131 | 2.922±0.087 |
| 11.621 | Glyceric acid (3TMS) | R | 1344 | 1321 | 0.031±0.001 | 0.035±0.007 | - | 0.042±0.005 |
| 13.6805 | Malic acid (3TMS) | RA | 1497 | 1477 | 36.794±4.394 | 8.304±1.347 | 4.556±0.625 | 3.698±0.233 |
| 16.6901 | Aconitic acid, (E)- (3TMS) | R | 1730 | 1732 | 0.040±0.012 | - | - | - |
| 17.3337 | Shikimic acid (4TMS) | R | 1843 | 1791 | 22.779±3.973 | 8.917±0.939 | 5.623±0.910 | 3.304±0.164 |
| 17.4578 | Citric acid (4TMS) | RA | 1845 | 1803 | 97.889±25.133 | 30.996±4.105 | 14.071±0.920 | 6.994±3.53 |
| 17.894 | Quininic acid (5TMS) | R | 1851 | 1845 | 7.406±2.225 | 3.086±0.377 | 2.049±0.407 | 1.424±0.09 |
| 19.817 | Palmitic Acid (TMS) | RA | 2050 | 2040 | 0.036±0.063 | 0.084±0.065 | 0.111±0.045 | 0.265±0.009 |
| 21.3538 | Linoleic Acid (TMS) | RA | 2212 | 2203 | - | 0.029±0.031 | - | 0.074±0.003 |
| 21.4086 | Oleic Acid, (Z)- (TMS) | R | 2218 | 2210 | - | 0.016±0.015 | - | 0.037±0.014 |
|  | **Total organic acid** |  |  |  | **165.56±35.82^a^** | **52.101±6.71^b^** | **27.297±2.05^b^** | **19.068±4.07 ^b^** |
| 10.8418 | Glycerol (3TMS) | RA | 1289 | 1264 | - | 5.755±1.169 | 10.120±1.358 | 32.261±1.791 |
| 13.9188 | meso-Erythritol (4TMS) | RA | 1501 | 1496 | - | - | - | 0.114±0.013 |
| 14.2973 | L-Threitol (4TMS) | R | 1510 | 1526 | 0.138±0.022 | 0.114±0.027 | 0.075±0.014 | 0.065±0.005 |
| 16.2244 | Xylitol (5TMS) | RA | 1741 | 1691 | 0.053±0.002 | - | - | - |
| 16.3828 | D-(+)-Arabitol (5TMS) | RA | 1710 | 1704 | - | 0.643±0.112 | 0.853±0.124 | 4.649±0.17 |
| 16.4477 | Adonitol (5TMS) | A | - | 1710 | - | - | 0.039±0.005 | 0.156±0.025 |
| 16.6355 | Ribitol (5TMS) | R | 1747 | 1703 | 0.120±0.024 | - | - | - |
| 18.7006 | D-Mannitol (6TMS) | RA | 1964 | 1924 | - | 0.187±0.015 | 0.131±0.026 | 0.101±0.004 |
| 20.2538 | Myo-Inositol (6TMS) | RA | 2129 | 2083 | 19.640±2.629 | 8.509±0.879 | 5.935±0.862 | 3.528±0.174 |
|  | **Total sugar alcohol** |  |  |  | **19.950±2.64 ^a^** | **15.208±2.18 ^a^** | **17.154±1.92 ^a^** | **40.872±2.05^b^** |
| 14.1292 | L-5-Oxoproline (2TMS) | R | 1522 | 1513 | 0.039±0.009 | - | - | - |
| 14.2314 | γ-aminobutyric acid (3TMS) | RA | 1532 | 1521 | - | 0.053±0.004 | 0.005±0.008 | - |
|  | **Total amino acid** |  |  |  | **0.039±0.009 ^a^** | **0.053±0.004 ^a^** | **0.005±0.008^b^** | **-** |
| 14.5298 | D-(-)-Erythrose (3TMS) | R | 1536 | 1545 | 0.062±0.016 | 0.009±0.015 | 0.028±0.024 | - |
| 15.5739 | D-(+)-Xylose (4TMS) | RA | 1637 | 1633 | 0.278±0.058 | 0.068±0.009 | 0.046±0.011 | - |
| 15.7288 | D-Arabinose (4TMS) | RA | 1654 | 1647 | 0.062±0.014 | 0.044±0.01 | 0.036±0.007 | - |
| 15.8865 | D-Ribose (4TMS) | A | - | 1661 | 0.078±0.01 | 0.065±0.016 | 0.062±0.014 | 0.140±0.017 |
| 16.4249 | D-(-)-Rhamnose (4TMS) | R | 1706 | 1708 | 0.036±0.008 | - | - | - |
| RT | Compound Name | ID^a^ | RI Lib | RI Cal^b^ | RM | B1 | B2 | MBC |
| 18.0129 | D-Fructose (5TMS) | RA | 1875 | 1856 | 169.558±11.14 | 38.826±3.211 | 1.018±0.135 | 200.419±8.341 |
| 18.2116 | D-Mannose (5TMS) | A | - | 1875 | 0.914±0.247 | 0.235±0.059 | 0.154±0.03 | 0.337±0.35 |
| 18.2871 | D-Glucose (5TMS) | A | - | 1883 | 81.791±9.867 | 13.756±1.438 | 0.357±0.098 | 125.713±10.05 |
|  | **Total monosaccharide** |  |  |  | **252.78±21.28 ^a^** | **53.003±3.53^b^** | **1.7±0.29^c^** | **326.609±18.30^d^** |
| 14.7497 | Tyrosol (2 TMS) | R | 1569 | 1564 | - | 0.021±0.019 | - | 0.032±0.028 |
|  | **Total phenolic compound** |  |  |  | **-** | **0.021±0.019 ^a^** | **-** | **0.032±0.028 ^a^** |
| 15.2376 | D-(-)-Ribofuranose (4TMS) | R | 1624 | 1604 | 0.118±0.028 | - | - | 0.078±0.01 |
| 16.9003 | L-(-)-Sorbofuranose (5TMS) | R | 1779 | 1752 | 0.170±0.004 | 0.058±0.005 | - | 0.154±0.033 |
| 16.9809 | D-(-)-Tagatofuranose (5TMS) | R | 1801 | 1759 | 0.123±0.051 | - | - | 1.111±0.109 |
| 19.0301 | β-D-Glucopyranose (5TMS) | R | 1931 | 1957 | - | - | - | 0.025±0.023 |
|  | **Total furanose & pyranose** |  |  |  | **0.411±0.075 ^a^** | **0.058±0.005 ^b^** | **-** | **1.369±0.148^c^** |
| 24.7506 | Sucrose (TMS) | A | - | 2618 | 7.568±0.944 | 0.298±0.07 | 0.039±0.014 | 365.108±56.34 |
| 25.5389 | Maltose (8TMS) | RA | 2733 | 2724 | - | 0.091±0.021 | - | 0.206±0.018 |
|  | **Total disaccharide** |  |  |  | **7.568±0.944^a^** | **0.389±0.09^a^** | **0.039±0.014^a^** | **365.314±56.32^b^** |

^a^Identification method: R, mass spectrum and RI agree with NIST14 library; A, mass spectrum and RI agree with authentic compound; RA, mass spectrum and RI agree with NIST14 library and mass spectrum with authentic compound.

^b^Retention index (RI) bases on DB-WAX capillary column using a series of alkanes between C_7_-C_40_.

All values are the mean ± SD (n=3) and different letter indicates a significant difference (Tukey HSD, p<0.05).

**Supplementary Table 3.** Volatile metabolites alteration of raw mango (RM), after brining1 (B1), after brining2 (B2), and final product (MBC).

|  | Log2(FC) | | |
| --- | --- | --- | --- |
| Metabolite | B1 to RM | B2 to B1 | MBC to B2 |
| **Alcohol** |  |  |  |
| Phenylethyl Alcohol | 26.554 | - | - |
| Isopentyl alcohol | 24.935 | - | 1.7133 |
| Isobutyl alcohol | - | - | 2.1967 |
| Isopropyl alcohol | 1.6286 | - | - |
| **Ester** |  |  |  |
| Acetic acid, 2-phenylethyl ester | 26.158 | - | - |
| Isopentyl acetate | 22.715 | - | 4.5117 |
| Ethyl 9-hexadecenoate | - | - | 20.868 |
| Tetradecanoic acid, ethyl ester | 20.428 | - | - |
| Dodecanoic acid, ethyl ester | - | 20.226 | 1.9532 |
| Hexadecanoic acid, ethyl ester | - | - | 20.173 |
| Ethyl Acetate | 3.9531 | -1.2452 | 3.0787 |
| Isopropyl myristate | - | - | 1.1947 |
| **Organic acid** | |  |  |
| Acetic acid | 21.994 | -1.0017 | - |
| **Ketone** |  |  |  |
| 5-Hepten-2-one, 6-methyl- | 21.111 | - | - |
| 6-Methyl-3,5-heptadiene-2-one | 2.1622 | -1.0314 | - |
| **Monoterpene & monoterpenoids** | | | |
| Neryl Acetate | 2.5956 | - | - |
| trans-Pinocarveol | 2.2333 | - | - |
| Geranylacetone | 1.0729 | - | - |
| L-Limonene | -1.0036 | - | - |
| γ-Terpinene | -1.4719 | -1.8506 | -19.112 |
| (+)-4-Carene | -19.868 | - | - |
| α-Phellandrene | -21.233 | - | - |
| trans-Ocimenol | - | -1.053 | - |
| β-Damascenone | - | -20.338 | 18.779 |
| **Sesquiterpene & sesquiterpenoids** | | | |
| Ledane | 1.7819 | - | - |
| α-Cubebene | 1.2551 | -21.211 | - |
| trans-Calamenene | -1.2972 | - | -19.667 |
| Germacrene B | -1.6175 | - | -20.057 |
| (-)-Aristolene | -2.0762 | -19.648 | - |
| Viridiflorene | -2.3703 | - | -19.989 |
| β-Elemene | -19.533 | - | - |
| Copaene | -25.155 | 23.168 | - |
| γ-Cadinene | - | 1.2983 | -1.659 |
| β-Ylangene | - | -1.0678 | - |
| β-Copaene | - | -1.1624 | - |
|  | Log2(FC) | | |
| Metabolite | B1 to RM | B2 to B1 | MBC to B2 |
| β-Gurjunene | - | -1.2953 | - |
| **Furan** |  |  |  |
| trans-Linalool oxide (furanoid) | 1.7249 | -3.2115 | - |
| 3-Methyl-2-(2-methyl-2-butenyl)-furan | - | - | 1.2966 |
| **Aldehyde** |  |  |  |
| Hexanal | 1.215 | -1.4005 | 2.2429 |
| 2-Octenal, (E)- | - | -1.1154 | - |
| **Aromatic compound** | | |  |
| α-Pyronene | -20.114 | - | - |
| m-Cymenene | -21.215 | - | - |

**Supplementary Table 4.** Non-volatile metabolites alteration of raw mango (RM), after brining1 (B1), after brining2 (B2), and final product (MBC).

|  | Log2(FC) | | |
| --- | --- | --- | --- |
| Metabolite | B1 to RM | B2 to B1 | MBC to B2 |
| **Sugar alcohol** | |  |  |
| Glycerol (3TMS) | 25.881 | - | 1.6726 |
| D-(+)-Arabitol (5TMS) | 22.72 | - | 2.446 |
| D-Mannitol (6TMS) | 20.939 | - | - |
| Myo-Inositol (6TMS) | -1.2068 | - | - |
| Xylitol (5TMS) | -18.991 | - | - |
| Ribitol (5TMS) | -20.168 | - | - |
| Adonitol (5TMS) | - | 18.562 | 1.9849 |
| meso-Erythritol (4TMS) | - | - | 20.047 |
| **Disaccharide** | |  |  |
| Maltose (8TMS) | 19.893 | -19.723 | 20.909 |
| Sucrose (TMS) | -4.665 | -2.9186 | 13.176 |
| **Amino acid** | |  |  |
| γ-aminobutyric acid (3TMS) | 19.111 | -3.5415 | - |
| L-5-Oxoproline (2TMS) | -18.568 | - | - |
| **Organic acid** | |  |  |
| Linoleic acid (TMS) | 18.224 | -18.054 | 19.426 |
| Oleic acid, (Z)- (TMS) | 17.365 | -17.196 | 18.418 |
| Succinic acid (2TMS) | 3.3465 | - | 2.0637 |
| Palmitic acid (TMS) | 1.1966 | - | 1.255 |
| Quininic acid (5TMS) | -1.2631 | - | - |
| Shikimic acid (4TMS) | -1.3531 | - | - |
| Citric acid (4TMS) | -1.6591 | -1.1394 | -1.0085 |
| Malic acid (3TMS) | -2.1475 | - | - |
| Phosphoric acid (3TMS) | -2.9689 | - | -1.6289 |
| Aconitic acid, (E)- (3TMS) | -18.586 | - | - |
| Lactic acid (2TMS) | - | 19.648 | - |
| Glyceric acid (3TMS) | - | -18.353 | 18.612 |
| Propanedioic acid (2TMS) | - | - | 19.025 |
| **Phenolic** |  |  |  |
| Tyrosol (2TMS) | 17.787 | -17.618 | 18.213 |
| **Furanose & pyranose** | | |  |
| L-(-)-Sorbofuranose (5TMS) | -1.5437 | -19.083 | 20.491 |
| D-(-)-Tagatofuranose (5TMS) | -19.211 | - | 22.339 |
| D-(-)-Ribofuranose (4TMS) | -20.151 | - | 19.499 |
| β-D-Glucopyranose (5TMS) | - | - | 17.871 |
| **Monosaccharide** | |  |  |
| D-Mannose (5TMS) | -1.9594 | - | 1.1346 |
| D-(+)-Xylose (4TMS) | -2.0286 | - | -17.593 |
| D-Fructose (5TMS) | -2.1267 | -5.2527 | 7.6206 |
|  | Log2(FC) | | |
| Metabolite | B1 to RM | B2 to B1 | MBC to B2 |
| D-Glucose (5TMS) | -2.5719 | -5.2681 | 8.4601 |
| D-(-)-Erythrose (3TMS) | -2.8557 | 1.6997 | -17.862 |
| D-Ribose (4TMS) | - | - | 1.1701 |
| D-Arabinose (4TMS) | - | - | -18.219 |
| D-(-)-Rhamnose (4TMS) | -18.431 | - | - |
